# Notes and Notices

**Published:** 1884-11

**Authors:** 


### NOTES AND NOTICES.

WANTED.—Numbers four and five, volume one, of this journal; address this office.

REMOVAL.—The office of this journal has been removed to No. 129 South Thirteenth Street. Correspondents will please take notice.

EVEN AN EARTHQUAKE POWERLESS.—Hardly had the Faraday laid the new Atlantic cable before it was broken by an earthquake; yet we feel sure no earthquake could shake a delinquent subscriber sufficiently to cause him to break up his debts.

NEW PARTNERSHIP.—The co-partnership heretofore existing between Dr. G. W. Barnes and Dr. E. A. Clarke, of San Diego, California, has been dissolved by mutual consent, Dr. Clarke retiring. A new partnership has been formed between Dr. Barnes and Morgan, of same city.

"ECLECTIC" MEDICAL EDUCATION.—Two eclectic physicians testified before a coroner's jury; one stated the normal healthy temperature to be about seventy-six or eighty degrees, but might be one hundred and forty! The other was not altogether clear, but believed seventy or eighty degrees to be about fair, and that it was higher in the Southern States. He took the temperature by a *barometer!* Some of our homœopathic medical schools could scarcely graduate more ignorant men than these are!

"MELLIN'S FOOD.—This preparation is, in fact, an excellent attempt to give the extractive and soluble portion of Liebig's food, without the cellular and indigestible part of the meal. In other preparations of this class this was partially avoided, but not wholly so, by straining. There is no evidence of starch remaining in this preparation, it having been all converted into grape-sugar and dextrine, and there is no reason to believe that it is prepared from anything but malt and wheat. As a food for delicate infants there can be no question as to its great value."—*Medical Press and Circular, London.*

FOOD EXTRACTS.—The value of Murdock's Liquid Food is fairly illustrated in the following case—a friend of ours: This lady suffered from general debility, the outcome of a residence in a malarious district, greatly intensified by unskilled treatment. Under homœopathic care and change of residence some improvement was noticeable, but the debility continued, notwithstanding close study of the case; a normal weight of one hundred and eighteen pounds was reduced to ninety-six, when Murdock's Food was prescribed, and in three months health was re-established, and a gain in weight of twenty-five pounds resulted from its continued use for some six months.—*Am. Hom.*

CHOLERA MICROBES.—Dr. Gregg, of Buffalo, New York, has long disputed the theory that the so-called "germs" cause the diseases with which they are found. Now, we find the following, confirmatory of Dr. Gregg's views: "Messrs. Roux and Strauss, two eminent French surgeons, now practicing in the hospitals at Toulon, and who studied cholera thoroughly last year in Egypt, have made an official report, declaring that they find the microbe to be the result, rather than the germ, of cholera. In certain 'foudroyant' cases (*i. e.*, those in which death comes most quickly, unaccompanied by vomiting or dejections) they have found no microbe at all; while in others, the number of bacilli is in proportion to the duration of the disease.

"They state that similar microbes are generated in the intestines by typhoid fever and other zymotic diseases, and that they are found by myriads in water, which, being drunk, does not create cholera. Animals have been fed and inoculated with bacilli taken from the alimentary canal of diseased cholera, patients without producing any effect whatever."

